# Extraction and Concentration of *Spirulina* Water-Soluble Metabolites by Ultrafiltration

**DOI:** 10.3390/plants13192770

**Published:** 2024-10-03

**Authors:** Claudia Salazar-González, Carolina Mendoza Ramos, Hugo A. Martínez-Correa, Hugo Fabián Lobatón García

**Affiliations:** 1 Tecnología en Gestión Gastronómica, Universitaria Agustiniana, Bogotá 110831, Colombia; claudia.salazar@uniagustiniana.edu.co; 2 Facultad de Ciencias Biológicas, Benemérita Universidad Autónoma de Puebla (BUAP), Puebla 72570, Mexico; carolina.mendozara@alumno.buap.mx; 3 Departamento de Ingeniería, Facultad de Ingeniería y Administración, Universidad Nacional de Colombia Sede Palmira, Palmira 763531, Colombia; hamartinezco@unal.edu.co; 4 Vicerrectoría de Investigaciones, Universitaria Agustiniana, Bogotá 110831, Colombia

**Keywords:** chlorophylls, conventional extraction, phycocyanin, protein, *Spirulina*, total carotenoids, ultrasound-assisted extraction (UAE)

## Abstract

*Spirulina* (*Arthospira platensis*) is known for its rich content of natural compounds like phycocyanin, chlorophylls, carotenoids, and high protein levels, making it a nutrient-dense food. Over the past decade, research has aimed to optimize the extraction, separation, and purification of these valuable metabolites, focusing on technologies such as high-pressure processing, ultrasound-assisted extraction, and microwave-assisted extraction as well as enzymatic treatments, chromatographic precipitation, and membrane separation. In this study, various extraction methods (conventional vs. ultrasound-assisted), solvents (water vs. phosphate buffer), solvent-to-biomass ratios (1:5 vs. 1:10), and ultrafiltration (PES membrane of MWCO 3 kDa, 2 bar) were evaluated. The quantities of total protein, phycocyanin (PC), chlorophyll a (Cla), and total carotenoids (TCC) were measured. The results showed that ultrasound-assisted extraction (UAE) with phosphate buffer at a 1:10 ratio yielded a metabolite-rich retentate (MRR) with 37.0 ± 1.9 mg/g of PC, 617 ± 15 mg/g of protein, 0.4 ± 0.2 mg/g of Cla, and 0.15 ± 0.14 mg/g of TCC. Water extraction in the concentration process achieved the highest concentrations in MRR, with approximately 76% PC, 92% total protein, 62% Cla, and 41% TCC. These findings highlight the effective extraction and concentration processes to obtain a metabolite-rich retentate from *Spirulina* biomass, reducing the volume tenfold and showing potential as a functional ingredient for the food, cosmetic, and pharmaceutical industries.

## 1. Introduction

The field of functional products is currently focused on identifying natural products and metabolites with exceptional properties, particularly those that are beneficial for nutrition and health. Among the sources rich in valuable metabolites, *Spirulina* (previously known as *Arthospira platensis*, genus Limnospira [1]) is recognized for its high content of various natural molecules. *Spirulina* boasts a significant protein content (up to 70%), including phycocyanin, carbohydrates, lipids such as essential amino acids and polyunsaturated fatty acids, pigments (phycocyanin, chlorophylls, and carotenoids), and minerals, many of which offer notable nutritional and health benefits [2,3].

Phycocyanin (PC), a blue pigment belonging to the phycobiliprotein (PBP) family, is the most extensively studied metabolite due to its potential functional activities, including antioxidant, antimicrobial, anticarcinogenic, anti-inflammatory, and anti-anemic properties, among others [3,4,5]. However, chlorophyll and carotenoids also exhibit promising functional activities, positioning these compounds as potential ingredients in the food, cosmetic, and pharmaceutical industries. In the last few years, phycocyanin has gained attention as an ingredient in the cosmeceutical sector.

Given its multiple biological activities and potential applications, the past decade has seen numerous studies focused on the extraction and concentration processes of these compounds. Extraction methods have included conventional extraction (CE), freeze–thaw, pulsed electric field, high-pressure processing, ultrasound-assisted extraction (UAE), and microwave-assisted extraction [3,5,6,7,8,9]. For concentration and separation processes, techniques such as precipitation with ammonium sulfate, enzymatic treatments, chromatographic precipitation, and membrane separation (MS) are widely used [3,10,11]. Among these, membrane separation processes stand out due to their environmental and cost-effective advantages as they do not require additional water and involve economical equipment compared to enzymatic or chromatographic methods.

Among membrane separation processes, ultrafiltration (UF) has emerged as a promising technology due to its ability to retain large molecules and proteins, which is crucial for the separation and purification of proteins and phycocyanin in *Spirulina* strains. Several studies have employed UF for this purpose. For instance, Balti et al. [6] utilized diafiltration membranes with molecular weight cut-offs of 50 kDa, 150 kDa, 300 kDa, and 0.2 µm, finding that filtration using the 0.2 µm membrane resulted in over 89% recovery of phycocyanin A in the retentate and phycocyanin C in the permeate. This pore-size membrane facilitated the production of blue-colored permeates.

Menegotto et al. [11] investigated various ultrafiltration membranes and operational conditions to concentrate and separate proteins from *Spirulina.* They determined that a hollow fiber membrane with a 50 kDa cut-off operating at a tangential flow of 1.5 bar combined with diafiltration could produce a protein concentrate containing 142 mg/L of phycocyanin, 90% antioxidant activity, and proteins with molecular weights ranging from 5 to 15 and 100 kDa. More recently, Kurpan et al. [12] achieved a phycocyanin recovery of approximately 54%, with a concentration of 1.1 ± 0.2 mg/mL PC, purity of 1 ± 0.2, and antioxidant activity of 84 ± 6 ppm ascorbic-acid-equivalent using a membrane with a 50 kDa molecular weight cut-off.

These studies demonstrate the potential of membrane filtration for the separation and purification of proteins and phycocyanin. However, to the best of our knowledge, the use of small-pore membranes in ultrafiltration to obtain a metabolite-rich retentate (MRR) containing protein, phycocyanin, chlorophyll, and carotenoids from *Spirulina* biomass remains underexplored. Therefore, the objective of this study was to evaluate different factors, namely the extraction method, solvent, and solvent-to-biomass ratio in the extraction and concentration of water-soluble metabolites from *Spirulina* biomass using ultrafiltration as an alternative to precipitation via salting out with ammonium sulfate.

## 2. Results

### 2.1. Extraction

The primary aim of this study was to achieve a high yield in the extraction of water-soluble metabolites from *Spirulina* biomass. To enhance the quantities of these compounds, the extraction strategy focused on evaluating and selecting critical factors that influence the efficiency of cell disruption and metabolite recovery, namely the extraction method, solvent, and biomass-to-solvent ratio. Preliminary assays were conducted to assess the effects of temperature and extraction time. The results of the extraction experimental design are presented in Table 1. For chlorophyll a and total carotenoids, no significant differences were found. However, statistically significant differences were observed for phycocyanin content, protein concentration, and purity index with respect to the extraction method, solvent, and biomass-to-solvent ratio.

### 2.2. Ultrafiltration Process (UF)

For the ultrafiltration (UF) concentration process, two treatments were selected (one with water and one with buffer) to evaluate the solvent’s effect on the concentration. According to statistical analysis, there were no significant differences between the two biomass/solvent ratios used. Therefore, UAE_Buffer_1:10 was selected for the buffer, and CE_H_2_O_1:10 was selected for the water. These treatments were chosen because they demonstrated good PC content extraction and required lower quantities of biomass, thus reducing process costs.

This study focused on the concentration of *Spirulina* metabolites via ultrafiltration (UF). The evaluation encompassed two extracts obtained through different extraction methods and solvents, and their performance was analyzed in terms of phycocyanin (PC), total protein, purity index (PI), chlorophyll a (Cla), and total carotenoids (TCC). The results, presented in Table 2, demonstrate the effectiveness of UF in concentrating *Spirulina* metabolites.

## 3. Discussion

### 3.1. Extraction

#### 3.1.1. Phycocyanin

Regarding temperature, the phycocyanin content was 11.9 ± 1.6 mg/g at 18 °C and 23.4 ± 3.0 mg/g at 28 °C, showing statistically significant differences. Higher temperatures enhance metabolite extraction due to increased molecular motion, which facilitates greater contact between the biomass and solvent. Additionally, high temperatures improve solvent penetration into the matrix, reduce solvent viscosity, and increase the diffusivity of bioactive compounds during extraction. Increasing the solvent temperature also enhances its solvating power, allowing more phycocyanin to dissolve and thereby improving the extraction yield [13]. Based on these results, the experimental design was conducted at 28 °C to optimize PC extraction.

The maximum PC yield obtained was 38.3 ± 1.4 mg/g (3.83%) using conventional extraction with water as the solvent, a 1:5 *w*/*v* ratio, and an extraction time of 18 h. These results align with the behavior of mass transfer processes, which are characterized by two phases: (1) an initial rapid diffusion of compounds (washing) and (2) a slower extraction phase characterized by a diffusive mechanism, involving the transfer of solutes from the interior of the particles to the solvent. The washing phase is marked by the rapid penetration of the solvent into the solid structure, followed by the dissolution of a fraction of the compounds in the solvent. The compounds extracted in this stage are assumed to be dispersed on the external surface of the matrix and dissolve almost instantaneously. In the diffusive stage, the transport of compounds into the solvent occurs slowly, driven solely by the concentration gradient. Compounds such as phycocyanin, chlorophyll, and carotenoids must overcome interactions binding them to the matrix and diffuse into the solvent [14]. This gradient is typically enhanced by thermal treatments or by increasing the time or contact surface of the matrix with the solvent.

Extended contact times facilitate the second extraction step, resulting in higher concentrations after 18 h of extraction compared to 6 h (29.0 ± 2.3 mg/g PC). Although statistically significant differences were observed, extending the extraction time does not substantially improve phycocyanin extraction to justify increased process costs. Therefore, a 6-h extraction time was selected for the conventional process in the experimental design.

In the study by Brião et al. [7], extractions were performed using water (freeze–thaw, ratio 1:30) and buffer (ratio 1:100) as solvents under conventional extraction conditions. Their results showed concentrations of 47.94 ± 0.46 mg/g PC and 58.54 ± 0.26 mg/g PC for water and buffer extractions, respectively. These values are higher than those obtained in the present study. The differences can be attributed primarily to the freeze–thaw process, which intensively disrupts the cells, and the extended extraction time in the buffer process (21 h), which allowed more PC extraction during the diffusive stage. This is comparable to the 18-h treatment in our study, which yielded the highest PC content.

In another study using UAE with a ratio of 1:30, the crude extract contained 131 mg/L PC, which falls within the range of the results obtained in the present work (46–380 mg/L). The higher amounts obtained in our study are primarily due to the shorter extraction time (5 min) compared to the reference (35 min) by Menegotto et al. [11]. Extended extraction times in ultrasound-assisted extraction (UAE) typically result in metabolite degradation due to the temperature increase induced by the ultrasonic waves.

The observed differences regarding the solvent choice (water or buffer) are primarily due to the stability of phycocyanin with sodium chloride at a pH close to 7.0. It has been reported that sodium chloride effectively preserves phycocyanin at this pH [15]. In terms of the extraction method, conventional extraction (CE) yielded better results when water was used as the solvent, likely due to the longer extraction time compared to UAE (3 h). However, when buffer was used, there were no statistically significant differences between the two extraction methods.

The superior results for UAE with the buffer can be attributed to the composition of the buffer solution and its effects during UAE. First, the salts used in the buffer maintained the pH at around 7.0 and acted as preservatives for phycocyanin. Second, the salts enhanced the cavitation phenomenon in UAE. When ultrasound waves pass through the medium, they displace molecules in the solution and generate pressure that overcomes the intermolecular forces, creating cavitation bubbles in the solvent [14,16]. The formation, expansion, and implosion of these microbubbles, known as acoustic cavitation [17], results in high-velocity jets that impact the biomass, causing cell disruption (liquid micro-jets and shockwave effects). This improves solvent penetration into the cells and increases the mass transfer of phycocyanin, chlorophyll, and carotenoids to the buffer. Additionally, the release of large amounts of energy, high temperature (5000 K), and pressure (1000–2000 atm) points, along with the conductivity of the salts in the medium, further enhance the compound extraction [18].

Based on the maximum quantity of phycocyanin in the biomass (44.3 ± 6.6 mg/g), the extraction yields ranged from 17% to 83% for UAE_H_2_O_1:5 and UAE_Buffer_1:10, respectively. These results suggest that the most effective extraction processes are those using buffer as the solvent.

#### 3.1.2. Protein and Purity Index (PI)

During the extraction process from *Spirulina* biomass, not only phycocyanin but also other proteins are extracted. Table 1 reports the total protein content and PI. The total protein content in the biomass was 707 ± 46 mg/g, with extraction yields ranging from 38% to 87% for UAE_H_2_O_1:5 and UAE_Buffer_1:10, respectively. As expected, the protein content was higher compared to PC, resulting in a low PI (<1.0). A PI of ≥0.7 indicates food-grade PC [5], thus only extractions using a buffer with a ratio of 1:10 *w*/*v* achieved food-grade PC status.

The PI reported in other studies varies from 0.3 to 0.74, depending on the extraction methods used [7,8]. Compared to Brião et al. [7], the present study obtained higher PIs, possibly due to the freeze–thaw process extracting all proteins from the biomass. In the study by Nisticò et al. [8], water extraction over 24 h resulted in concentrations of 0.23 mg/mL and a PI of 0.74 (ratio 1:20). Although the PC amount was lower, the higher PI can be attributed to fewer extracted proteins and cell debris, which increased the PI in the present study (0.44 mg/mL and PI of 0.6, CE_H_2_O_1:5, t: 18 h).

#### 3.1.3. Chlorophyll a (Cla) and Total Carotenoid Content (TCC)

The results for chlorophyll a and total carotenoids indicate that conventional extraction (CE) with water is the most effective process for recovering these valuable compounds (0.6–0.9 mg/g Cla and 0.25–0.50 mg/g TCC) compared to extractions using buffer (0.3–0.6 mg/g Cla and 0.10–0.15 mg/g TCC). However, the recovery was only 19% for chlorophyll a and 84% for carotenoids in the water extractions compared to the biomass characterization (4.6 mg/g Cla and 0.59 mg/g TCC) determined in an analytical way.

These results are consistent with those reported in the literature (0.28–4.43 mg/g) for total carotenoids [19]. According to the authors, 20–37% of the carotenoids are all-trans-zeaxanthin, a xanthophyll with higher water solubility than carotenes. It is likely that zeaxanthin and other xanthophylls were the dominant carotenoids in *Spirulina* biomass, explaining the high recovery rate. Despite good results in extracting chlorophyll and carotenoids, the majority of carotenoids remain in the biomass due to their lipophilic nature.

### 3.2. Extraction Ultrafiltration Process (UF)

The results in Table 3 demonstrate the effectiveness of UF in concentrating *Spirulina* metabolites. This is evidenced by the high concentrations of PC, total protein, PI, Cla, and TCC in the retentate, with minimal quantities of these compounds present in the permeate. These findings align with those reported by Menegotto et al. [11] and are attributed to the small pore size of the membrane used (MWCO 3 kDa), which effectively retains the valuable compounds.

**Figure 1:**
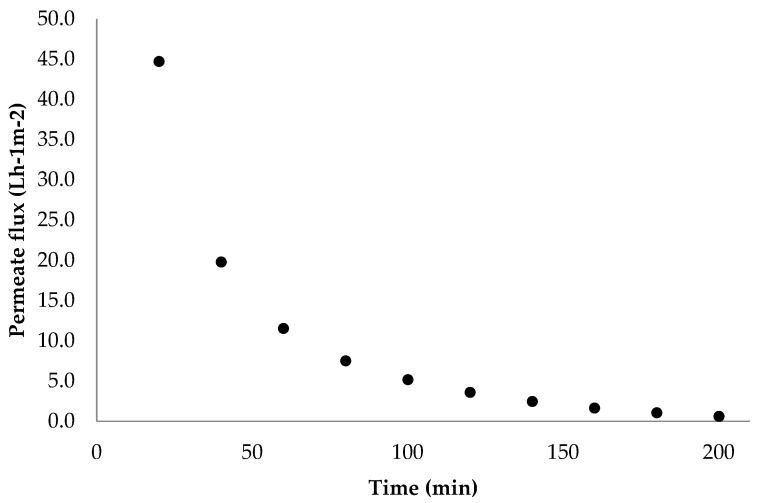
Permeate flux during the ultrafiltration of crude extract.

The ultrafiltration (UF) process exhibited behavior consistent with previous reports, showing a decrease in the permeate flow rate over the filtration time and a tenfold volume reduction (Figure 1) [5,8]. Initially (20–60 min), the flow rate decreased rapidly, followed by a slower reduction until 140 min, at which point the flux stabilized. According to Ho and Zydney [20], the fouling phenomenon occurs in two stages: pore blocking followed by cake layer formation. The first stage involves the adsorption of proteins and other molecules on the membrane surface, leading to a rapid decrease in the flow rate due to concentration polarization. The second stage involves membrane fouling and the subsequent formation of a cake layer, resulting in a gradual decline in the flow rate [7,21].

To evaluate the solvent performance in the UF process, this study investigated the characterization of the extract, retentate, and permeate fractions. Table 2 presents the phycocyanin content, total protein, purity index, chlorophyll a, and total carotenoid content in the crude extract, permeate, and retentate, referred to as metabolite-rich retentate (MRR). The data indicate that the retentate from both solvent extractions contained higher quantities of all the compounds (expressed in g/L or mg/L) compared to the crude extract used as the system feed. This finding demonstrates the ultrafiltration process’s efficacy in concentrating high-value metabolites from *Spirulina* biomass.

The green pigments from microalgae are typically discarded, with several studies concentrating on the blue fraction and methods for its purification. However, Alotaiby et al. [22] reported that green pigments possess significantly higher antioxidant values compared to phycocyanin. Therefore, the presence of chlorophyll in the metabolite-rich retentate (MRR) enhances its functional activity.

Water extraction yielded the highest concentration of compounds, resulting in approximately 94%, 92%, 62%, and 41% of phycocyanin (PC), total protein, chlorophyll a (Cla), and total carotenoid content (TCC), respectively, in the metabolite-rich retentate (MRR). This outcome may be attributed to the presence of salts in the buffer, which can drag some of the compounds into the permeate. This is supported by the observation that the permeate maintained a pH of around 7.0, indicating the presence of salts. Additionally, this permeate fraction could be utilized as an extraction solvent in subsequent processes due to its ability to maintain a pH of around 7.0. Regarding carotenoids, it is possible that they bind to high molecular weight molecules such as proteins, explaining the high recovery rate in the retentate, which aligns with the findings obtained by Paes et al. [23].

As previously mentioned, the purity index (PI) was low due to the substantial quantities of proteins recovered. Given that the aim of this study was to extract and concentrate water-soluble metabolites from *Spirulina* biomass, a small-pore membrane was employed. This choice facilitated the acquisition of a metabolite-rich retentate, which explains the lack of increase in the PI in the retained fraction.

**Figure 2:**
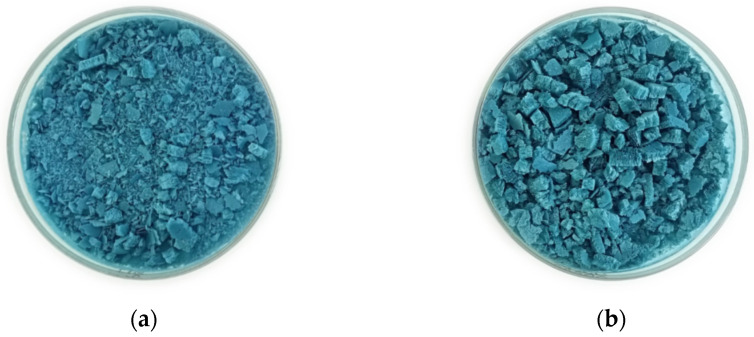
Lyophilized retentates (**a**) from buffer extraction and (**b**) from water extraction.

Based on the characterization of the extract, lyophilized retentate (Figure 2), and the mass balance, a decrease in the total mass obtained was evident. This reduction is attributed to losses during the process, including the membrane, and the filtration equipment. Nonetheless, the retentate remained rich in diverse metabolites and holds potential as a functional ingredient or raw material for subsequent fractionation processes. Additionally, the freeze-drying process offers a conservation advantage, as noted by Kurpan et al. [12] and Papalia et al. [2], due to the ease of protein degradation (within 2 days) and subsequent transport.

In summary, the ultrafiltration (UF) process offers several advantages, including a tenfold reduction in volume, the production of a metabolite-rich retentate from the crude extract, and reduced energy costs in the subsequent drying process. This study demonstrates that UF is a viable alternative to precipitation by salting out with ammonium sulfate, as the metabolite-rich retentate is concentrated in phycocyanin, which is the primary objective of this separation process. Moreover, UF eliminates the need for chemical reactants and the subsequent separation from the target metabolite, thereby reducing production costs [10,22].

## 4. Materials and Methods

### 4.1. Chemicals and Biomaterials

The biomass of *Spirulina* was obtained from a *Spirulina* farm situated in Cumaral, Meta, Colombia. Methanol of analytical grade was sourced from PanReac AppliChem. Sodium chloride (NaCl), potassium chloride (KCl), sodium phosphate monobasic (NaH_2_PO_4_), and potassium phosphate dibasic (K_2_HPO_4_) were obtained from Merck. The polyether sulfone ultrafiltration membrane was also acquired from Merck, Darmstadt, Germany.

### 4.2. Extraction Methods

#### 4.2.1. Experimental Design

For the extraction of metabolites, two methodologies were employed: conventional extraction (CE) and ultrasound-assisted extraction (UAE). The solvents utilized included distilled water and pH 7.4 phosphate buffer, which was prepared using sodium chloride (NaCl), potassium chloride (KCl), sodium phosphate monobasic (NaH_2_PO_4_), and potassium phosphate dibasic (K_2_HPO_4_). The experimental design is detailed in Table 3.

#### 4.2.2. Conventional Extraction (CE)

In the conventional extraction process, the procedure was performed by following the method described by Chaiklahan et al. [24], with some modifications. The biomass and solvent were combined using a stirring and heating plate, as outlined in the experimental design provided in Table 1. The mixture was maintained at 28 °C and stirred at 800 rpm for 6 h. After 3 h of extraction, the samples were centrifuged at 5000 rpm for 10 min. The supernatant was decanted, and the residual biomass was subjected to an additional extraction phase for another 3 h. The resulting fractions were then combined and analyzed. Each experimental condition was replicated in duplicate.

#### 4.2.3. Ultrasound-Assisted Extraction (UAE)

The extraction of metabolites was carried out following the method described by López et al. [25], with some modifications. To disrupt the cells within the biomass, the samples were initially agitated for 10 s and then subjected to ultrasonic treatment in a bath operating at 40 kHz for 10 min at full power. The temperature of the ultrasonic bath was meticulously monitored to ensure it did not exceed 30 °C. Following the ultrasonication process, the samples were incubated in darkness at room temperature for 1 h. After the extraction process, the samples were centrifuged at 5000 rpm for 10 min. The resulting supernatant was decanted, and the biomass was subjected to a secondary extraction with a 2-h standing period. The fractions obtained from both extraction steps were then combined and analyzed. Each experimental condition was performed in duplicate to ensure the reliability and reproducibility of the results.

### 4.3. Concentration (Ultrafiltration)

The crude extract was concentrated using ultrafiltration equipment, which comprised a 500 mL feed tank, a peristaltic pump, a pressure control valve, and a permeate tank. A polyethersulfone (PES) membrane module, featuring a molecular weight cut-off (MWCO) of 3 kDa and a membrane surface area of 0.02 m², was employed to concentrate the crude extract. The ultrafiltration was conducted at a pressure of 2 bar and at room temperature.

Following the concentration process, the membrane underwent a two-step cleaning procedure. The initial cleaning involved recirculating 150 mL of 5N NaOH solution. This was followed by a second cleaning step using 150 mL of distilled water. This cleaning protocol was designed to restore the membrane’s permeability to its optimal level.

### 4.4. Freeze-Drying

The drying process was conducted under full vacuum conditions, with the sample temperature maintained at −60 °C for 16 h using a Biobase BK-FD12P freeze-drier.

### 4.5. Quantification of Intracellular Compounds

The phycocyanin content (PC), total protein, purity index (PI), chlorophyll a (Cl_a_), and total carotenoid content (TCC) in the biomass were determined according to the protocol established by López et al. [25]. These parameters were also measured in the crude extract, permeate, and retentate using the corresponding protocols.

#### 4.5.1. Phycocyanin Content (PC)

The quantification of the phycocyanin content (PC) was conducted following an adapted protocol by López et al. [25]. An aliquot from the extract obtained in Section 2.2 (Extraction Methods) was measured using a UV-VIS spectrophotometer (Merck Spectroquant Prove 300) at wavelengths of 720 nm, 652 nm, and 615 nm. Dilutions were made if necessary. The phycocyanin content was quantified in g/L using Equation (1). All the measurements were performed in triplicate.

(1) $$PC\left(g/L\right)=\frac{{(A}_{615}-A_{720})-0.474\;*\;(A_{652}-A_{720})}{5.34}\;*\;DF$$

#### 4.5.2. Protein and Purity Index (PI)

Protein quantification was measured by absorbance at 280 nm and reported in mg/g using Equation (2). The purity index (PI) was calculated as the ratio of absorbances at 280 nm and 615 nm, following the methodology described by Abalde et al. [26].

(2) $$P\;\left(mg/g\right)=A_{280}\;*\;1.55\frac{V(mL)}{Biomass\;(g)}$$

(3) $$PI=\frac{A_{615}}{A_{280}}$$

#### 4.5.3. Chlorophyll a (Cla) and Total Carotenoid Content (TCC)

An aliquot of 200 µL was separated from the extract obtained in Section 2.2 (Extraction Methods) and diluted with 1300 µL of methanol, following the protocol by Balti et al. [6]. The mixture was agitated for 10 s and then kept in the dark at 45 °C for 1 h. Subsequently, the extract was centrifuged at 15,000 rpm for 5 min, and the absorbances of the supernatant were measured spectrophotometrically at 652 nm, 665 nm, and 470 nm. The chlorophyll a content (mg/g) was calculated using Equation (4) [27], and the total carotenoids (mg/g) were quantified using Equation (5) [28]. All the measurements were performed in triplicate.

(4) $${Cl}_{a}\left(\frac{mg}{g}\right)=12.9447\;*\;A_{665}\;*\;\frac{V(mL)}{Biomass(mg)}$$

(5) $$TCC\left(\frac{mg}{g}\right)=\frac{\left(1000\;*\;A_{470}-1.63\;*\;{Cl}_{a}(mg/L)\right)}{221}\;*\;\frac{V(mL)}{Biomass(mg)}$$

#### 4.5.4. Rejection

The rejection rates of phycocyanin, chlorophyll a, and carotenoids were calculated as the total mass of each metabolite in the retentate divided by the total amount available in the crude extract, expressed as a percentage.

### 4.6. Statistical Analysis

Descriptive statistical analysis was performed in Microsoft Excel 2016 (Microsoft, Redmond, WA, USA) to determine the averages and standard deviations for all the measurements. Analysis of variance (ANOVA) and Tukey tests at a 95% confidence level were conducted using Minitab.20^®^ software to evaluate the differences among the treatments.

## 5. Conclusions

The maximum phycocyanin (PC) yield obtained was 38.3 ± 1.4 mg/g (3.83%) using conventional extraction with water as the solvent, a biomass-to-solvent ratio of 1:5 *w*/*v*, and an extraction time of 18 h. These conditions resulted in an 83% extraction yield. Furthermore, the presence of chlorophyll and carotenoids in the crude extract highlights its potential applications in the food, cosmetic, and pharmaceutical industries due to antioxidant, antimicrobial, and other beneficial activities provided by phycocyanin, chlorophyll, and carotenoids.

The ultrafiltration (UF) processes were conducted with two treatments: UAE_Buffer_1:10 and CE_H_2_O_1:10. Both treatments produced a metabolite-rich retentate (MRR); however, water extraction achieved the highest concentrations in the MRR, obtaining approximately 94%, 92%, 62%, and 41% for PC, total protein, chlorophyll a (Cla), and total carotenoids (TCC), respectively. The UF processes demonstrated the ability to generate an MRR and reduce the volume by tenfold, which will subsequently decrease energy costs in the drying process.

## Figures and Tables

**Table 1:** Water-soluble metabolites (mg/g) from *Spirulina* (*Arthospira platensis*) biomass in the crude extract.

Treatment	PC (mg/g)	Protein (mg/g)	PI	Cl_a_ (mg/g)	TCC (mg/g)
CE_H_2_O_1:10	11.9 ^d^ ± 1.6	648 ± 195	0.3 ^d^ ± 0.003	0.9 ^a^ ± 0.50	0.50 ^a^ ± 0.43
T: 18 °C					
CE_H_2_O_1:10	23.4 ^c^ ± 3.0	557 ± 11	0.5 ^bcd^ ± 0.05	0.6 ^a^ ± 0.58	0.25 ^a^ ± 0.34
CE_H_2_O_1:5	29.0 ^bc^ ± 2.3	479 ± 50	0.7 ^ab^ ± 0.003	0.7 ^a^ ± 0.11	0.18 ^a^ ± 0.007
CE_H_2_O_1:5	38.3 ^a^ ± 1.4	531 ± 47	0.6 ^abc^ ± 0.2	1.5 ^a^ ± 0.72	0.36 ^a^ ± 0.12
t: 18 h					
UAE_H_2_O_1:10	9.0 ^d^ ± 0.7	600 ± 115	0.2 ^d^ ± 0.02	0.5 ^a^ ± 0.12	0.18 ^a^ ± 0.10
UAE_H_2_O_1:5	7.7 ^d^ ± 3.3	271 ± 27	0.3 ^cd^ ± 0.09	0.2 ^a^ ± 0.06	0.02 ^a^ ± 0.004
CE_Buffer_1:5	35.4 ^ab^ ± 0.8	541 ± 4.5	0.8 ^ab^ ± 0.03	0.6 ^a^ ± 0.20	0.14 ^a^ ± 0.07
UAE_Buffer_1:10	37.0 ^a^ ± 1.9	617 ± 15	0.6 ^ab^ ± 0.04	0.4 ^a^ ± 0.2	0.15 ^a^ ± 0.14
UAE_Buffer_1:5	36.1 ^a^ ± 0.03	416 ± 33	0.9 ^a^ ± 0.06	0.3 ^a^ ± 0.15	0.10 ^a^ ± 0.08

CE: conventional extraction; UAE: ultrasound-assisted extraction; PC: phycocyanin content; PI: purity index; Cla: chlorophyll a; TCC: total carotenoid content; 1:5: ratio biomass/solvent 1:5; 1:10: ratio biomass/solvent 1:10. Means that do not share a letter are significantly different.

**Table 2:** Metabolite characterization from *Spirulina* (*Arthospira platensis*) in crude extract, retentate, and permeate.

	Buffer Extraction				Water Extraction			
	Crude Extract	Retentate	Permeate	Rejection (%)	Crude Extract	Retentate	Permeate	Rejection (%)
V (mL)	341	32	305		333	35	280	
PC (g/L)	0.21	2.0			0.21	1.8		
**PC (mg)**	**71.6**	**65.2**	**0**	**91%**	**68.6**	**64.5**	**0**	**94%**
Protein (g/L)	3.57	32.7	1.0		4.05	35.5	0.62	
**Protein (mg)**	**1217**	**1047**	**306**	**86%**	**1350**	**1242**	**174**	**92%**
PI	0.6	0.6			0.6	0.6		
Chl_a_ (mg/L)	2.84	16			4.2	24.7		
**Chl_a_ (ug)**	**971**	**511**	**0**	**52%**	**1400**	**864**	**0**	**62%**
TCC (mg/L)	0.92	2.9			0.83	3.23		
**TCC (ug)**	**313**	**95**	**0**	**30%**	**277**	**113**	**6.3**	**41%**
**Salts (mg)**	**7843**	**784**		**10%**	**-**	**-**		
**RLio (mg)**		**993**				**1023**		

PC: phycocyanin content; PI: purity index; Chla: chlorophyll a; TCC: total carotenoid content; RLio: retentate lyophilized.

**Table 3:** Experimental design.

Extraction Method	Solvent	Ratio (*w*/*v*)
Conventional	Water	1:5
Conventional	Water	1:10
Ultrasound-assisted	Water	1:5
Ultrasound-assisted	Water	1:10
Conventional	Buffer	1:10
Ultrasound-assisted	Buffer	1:5
Ultrasound-assisted	Buffer	1:10

## Data Availability

The data supporting the findings of this study are available from the corresponding author upon reasonable request.

## References

[1] Bürck M., Ramos S.d.P., Braga A.R.C. (2024). Enhancing the Biological Effects of Bioactive Compounds from Microalgae through Advanced Processing Techniques: Pioneering Ingredients for Next-Generation Food Production. Foods.

[2] Papalia T., Sidari R., Panuccio M.R. (2019). Impact of Different Storage Methods on Bioactive Compounds in Arthrospira platensis Biomass. Molecules.

[3] Athiyappan K.D., Routray W., Paramasivan B. (2024). Phycocyanin from *Spirulina*: A comprehensive review on cultivation, extraction, purification, and its application in food and allied industries. Food Humanit..

[4] de Jesus Raposo M.F., de Morais R.M.S.C., de Morais A.M.M.B. (2013). Health applications of bioactive compounds from marine microalgae. Life Sci..

[5] de Amarante M.C.A., Corrêa Júnior L.C.S., Sala L., Kalil S.J. (2020). Analytical grade C-phycocyanin obtained by a single-step purification process. Process Biochem..

[6] Balti R., Zayoud N., Hubert F., Beaulieu L., Massé A. (2021). Fractionation of Arthrospira platensis (*Spirulina*) water soluble proteins by membrane diafiltration. Sep. Purif. Technol..

[7] Brião V.B., Sbeghen A.L., Colla L.M., Castoldi V., Seguenka B., de Oliveira Schimidt G., Costa J.A.V. (2020). Is downstream ultrafiltration enough for production of food-grade phycocyanin from Arthrospira platensis?. J. Appl. Phycol..

[8] Nisticò D.M., Piro A., Oliva D., Osso V., Mazzuca S., Fagà F.A., Morelli R., Conidi C., Figoli A., Cassano A. (2022). A Combination of Aqueous Extraction and Ultrafiltration for the Purification of Phycocyanin from Arthrospira maxima. Microorganisms.

[9] Martínez-Vega J.E., Villafaña-Estarrón E., Escalante F.M.E. (2023). Comparative Study of the Efficiency of Additives in the Extraction of Phycocyanin-C from Arthrospira maxima Using Ultrasonication. Molecules.

[10] Hockey J.T. (2022). Improving an Aqueous Two-Phase Process for C-Phycocyanin Extraction from *Spirulina*. Master’s Thesis.

[11] Menegotto A.L.L., Fernandes I.A., Colla L.M., Duarte J., Andrade M.Z., Abirached C., Franceschi E., Steffens J., Valduga E. (2020). Thermic and techno-functional properties of Arthrospira platensis protein fractions obtained by membrane separation process. J. Appl. Phycol..

[12] Kurpan D., Idà A., Körner F., Lauceri R., Rocculi P., Phillips R., Schievano A. (2023). Pilot-scale concentration and partial purification of food-grade phycocyanin from Arthrospira platensis via cross flow filtration: From biomass to final product. J. Appl. Phycol..

[13] Chan C.H., Yusoff R., Ngoh G.C., Grumezescu A.M., Holban A.M.B.T. (2017). Chapter 15—An Energy-Based Approach to Scale Up Microwave-Assisted Extraction of Plant Bioactives. Handbook of Food Bioengineering.

[14] Vinatoru M., Mason T.J., Calinescu I. (2017). Ultrasonically assisted extraction (UAE) and microwave assisted extraction (MAE) of functional compounds from plant materials. TrAC Trends Anal. Chem..

[15] Chaiklahan R., Chirasuwan N., Bunnag B. (2012). Stability of phycocyanin extracted from *Spirulina* sp.: Influence of temperature, pH and preservatives. Process Biochem..

[16] Chemat F., Rombaut N., Sicaire A.G., Meullemiestre A., Fabiano-Tixier A.S., Abert-Vian M. (2017). Ultrasound assisted extraction of food and natural products. Mechanisms, techniques, combinations, protocols and applications. A review. Ultrason. Sonochem..

[17] Rutkowska M., Namieśnik J., Konieczka P., Pena-Pereira F., Tobiszewski M.B.T. (2017). Chapter 10—Ultrasound-Assisted Extraction.

[18] Fu X., Belwal T., Cravotto G., Luo Z. (2020). Sono-physical and sono-chemical effects of ultrasound: Primary applications in extraction and freezing operations and influence on food components. Ultrason. Sonochem..

[19] Park W.S., Kim H.-J., Li M., Lim D.H., Kim J., Kwak S.-S., Kang C.-M., Ferruzzi M.G., Ahn M.-J. (2018). Two Classes of Pigments, Carotenoids and C-Phycocyanin, in *Spirulina* Powder and Their Antioxidant Activities. Molecules.

[20] Ho C.C., Zydney A.L. (2000). A Combined Pore Blockage and Cake Filtration Model for Protein Fouling during Microfiltration. J. Colloid Interface Sci..

[21] Zhang W., Ding L., Grimi N., Jaffrin M.Y., Tang B. (2017). Application of UF-RDM (Ultafiltration Rotating Disk Membrane) module for separation and concentration of leaf protein from alfalfa juice: Optimization of operation conditions. Sep. Purif. Technol..

[22] Alotaiby S., Zhao X., Boesch C., Sergeeva N.N. (2024). Sustainable approach towards isolation of photosynthetic pigments *from Spirulina* and the assessment of their prooxidant and antioxidant properties. Food Chem..

[23] Paes J., Da Cunha C.R., Viotto L.A. (2015). Concentration of lycopene in the pulp of papaya (*Carica papaya* L.) by ultrafiltration on a pilot scale. Food Bioprod. Process..

[24] Chaiklahan R., Chirasuwan N., Loha V., Tia S., Bunnag B. (2011). Separation and purification of phycocyanin from *Spirulina* sp. using a membrane process. Bioresour. Technol..

[25] López Mejía N., Martínez Correa H.A., Lobatón García H.F. (2024). Biostimulating activity of biomass extracts and supernatants from a culture of Arthrospira platensis enriched with L-tryptophan. J. Appl. Phycol..

[26] Abalde J., Betancourt L., Torres E., Cid A., Barwell C. (1998). Purification and characterization of phycocyanin from the marine cyanobacterium *Synechococcus* sp. IO9201. Plant Sci..

[27] Ritchie R.J. (2006). Consistent sets of spectrophotometric chlorophyll equations for acetone, methanol and ethanol solvents. Photosynth. Res..

[28] Lichtenthaler H.K. (1987). Chlorophylls and Carotenoids: Pigments of Photosynthetic Biomembranes. Methods Enzymol..

